# Modifications in Vaginal Microbiota and Their Influence on Drug Release: Challenges and Opportunities

**DOI:** 10.3390/pharmaceutics11050217

**Published:** 2019-05-06

**Authors:** Gerardo Leyva-Gómez, María L. Del Prado-Audelo, Silvestre Ortega-Peña, Néstor Mendoza-Muñoz, Zaida Urbán-Morlán, Maykel González-Torres, Manuel González-Del Carmen, Gabriela Figueroa-González, Octavio D. Reyes-Hernández, Hernán Cortés

**Affiliations:** 1 Departamento de Farmacia, Facultad de Química, Universidad Nacional Autónoma de México, Ciudad de México 04510, Mexico; ml.delprado@iim.unam.mx or luisa.delpradoa@gmail.com (M.L.D.P.-A.); mzum_1212@hotmail.com (Z.U.-M.); 2 Laboratorio de Posgrado en Tecnología Farmacéutica, FES-Cuautitlán, Universidad Nacional Autónoma de México, Cuautitlán Izcalli 54740, Mexico; 3 Laboratorio de Infectología, Instituto Nacional de Rehabilitación Luis Guillermo Ibarra Ibarra, Mexico City 14389, Mexico; silvestreortega@yahoo.com.mx; 4 Facultad de Ciencias Químicas, Universidad de Colima, Colima 28400, Mexico; nmendoza0@ucol.mx; 5 CONACyT-Laboratorio de Biotecnología, Instituto Nacional de Rehabilitación Luis Guillermo Ibarra Ibarra, Ciudad de México 14389, Mexico; mikegcu@gmail.com; 6 Instituto Tecnológico y de Estudios Superiores de Monterrey, Campus Ciudad de México 14380, Mexico; 7 Facultad de Medicina, Universidad Veracruzana, Ciudad Mendoza, Veracruz 94740, Mexico; manueldelcarmen@hotmail.com; 8 CONACyT-Laboratorio de Genómica, Dirección de Investigación, Instituto Nacional de Cancerología. Av. San Fernando 22, Tlalpan, Sección XVI, Ciudad de México 14080, Mexico; gabufg@gmail.com; 9 Laboratorio de Biología Molecular del Cáncer, UMIEZ, Facultad de Estudios Superiores Zaragoza, Universidad Nacional Autónoma de México, Ciudad de México 09230, Mexico; maiden_sp@yahoo.com.mx; 10 Laboratorio de Medicina Genómica, Departamento de Genética, Instituto Nacional de Rehabilitación Luis Guillermo Ibarra Ibarra, Ciudad de México 14389, Mexico

**Keywords:** vaginal drug delivery, vaginal microbiota, biofilm, drug release, hydrogels, pH sensitive hydrogels

## Abstract

Vaginal drug delivery represents an attractive alternative to achieve local and systemic effects due to the high contact surface exposed, the mucoadhesion of the epithelium, and the high innervation that facilitates the absorption of drugs into the bloodstream. However, despite the confinement of the vaginal cavity, it is an organ with a highly variable microenvironment. Mechanical alterations such as coitus, or chemical changes such as pH and viscosity, modify the release of drugs. In addition, changes in vaginal microbiota can influence the entire vaginal microenvironment, thus determining the disposition of drugs in the vaginal cavity and decreasing their therapeutic efficacy. Therefore, the influence of microorganisms on vaginal homeostasis can change the pre-established scenario for the application of drugs. This review aims to provide an explanation of normal vaginal microbiota, the factors that modify it, its involvement in the administration of drugs, and new proposals for the design of novel pharmaceutical dosage forms. Finally, challenges and opportunities directed toward the conception of new effective formulations are discussed.

## 1. Introduction

The vaginal cavity is a pivotal component of the female reproductive system; it extends from the cervix and uterus to the external genitalia (vulva). Currently, vaginal drug delivery is commonly employed for diverse purposes, including the treatment of neoplastic lesions, local infections, and for contraceptive purposes [1]. Although the vaginal area is generally considered for the administration of drugs with local activity, due to its diverse attributes, it may also be an attractive site for the delivery of compounds when a systemic effect is desired [2]. First, the vagina is an organ with high vascularity; it possesses a dense network of blood vessels, arteries, and lymphatic vessels. In addition, another relevant feature of the vagina is that drugs absorbed there neither undergo first-pass gastrointestinal degradation nor the hepatic first-pass effect. This is because the blood leaving the vagina reaches peripheral circulation via the dense venous plexus, which releases primarily into the internal iliac veins. Therefore, drugs with the ability to cross the vaginal mucosa could reach high concentrations in the bloodstream, which would improve the efficacy of medication compared with the same drug administered by oral route. In support of this statement, it has been reported that propranolol showed higher bioavailability after vaginal compared with oral administration. In a similar manner, misoprostol, a drug used to induce labor, was more effective when administered via the vagina compared with other routes [3].

However, some concerns have arisen concerning the acceptability of vaginal products, including possible systemic adverse effects and particularly, vaginal microbiota affecting the drug absorption [4]. Vaginal microbiota plays an essential role in the health state of the female reproductive system through competition with other microorganisms for nutrients and the secretion of substances such as lactic acid, which render the vaginal environment inhospitable to other potentially pathogenic microbes. Therefore, the design of formulations for intravaginal delivery should consider not causing negative effects on the vaginal environment and the balance of the normal microbiota [5]. An imbalance in normal microbiota may be produced by a variety of factors, such as hormonal changes, douching, medical treatments, and sexual activity, which may lead to pathological conditions such as bacterial vaginosis, aerobic vaginitis, and sexually transmitted infections. The functional consequences of these disorders may include increased vaginal enzymatic activity and the formation of bacterial biofilms, which could lead to alterations in the drug absorption. In this regard, although in recent years there has been increasing interest in the vaginal route for the administration of drugs, the impact of alterations in vaginal microbiota on drugs absorption has not received significant attention. 

The employment of inert and non-irritant materials in order to develop appropriate vaginal drug-delivery systems and improve the acceptability of vaginal medication has been proposed. Likewise, the development of mucoadhesive materials has been useful in preparing new or improved systems with better performance [6]. Mucoadhesive properties can be exploited to prepare films, micro- and nanoparticles, niosomes, and other carriers, with the main goal of prolonging contact time and intimate interaction with the vaginal mucosa, which would produce the continuous and prolonged delivery of the drug, preventing concentration fluctuations or the high peak concentrations observed with oral administration [6].

In this article, we describe the main factors that modify vaginal microbiota and the physicochemical changes in the vaginal environment produced by that modification. The main goal of this article is to provide an outlook on the role of alterations of the local microbiota on drug release administered by the vaginal route and to describe the most suitable formulation to maintain therapeutic efficacy under this condition.

## 2. Vaginal Microbiota

### 2.1. Composition of the Vaginal Microbiota

The term microbiota refers to the whole collection of microorganisms in a specific niche, such as the human gut, oral cavity, and vaginal area [7]. In this section, we will review the composition of vaginal microbiota in a health-associated state and what takes place when an alteration in the composition of the microorganisms, mainly alterations in bacterial communities, occurs (dysbiosis). 

In healthy women, the predominant microorganism in vaginal microbiota appears to be *Lactobacillus* spp. [8], and the more abundant species are *Lactobacillus crispatus*, *Lactobacillus gasseri*, *Lactobacillus iners*, and *Lactobacillus jensenii* [9,10]. However, other species exist at low proportions, such as *Bacteroides, Fusobacterium*, *Veillonela*, *Actinomycetes*, *Bifidobacterium*, *Peptococcus*, *Peptostreptococcus*, *Propionibacterium*, *Staphylococcus aureus*, *Staphylococcus epidermidis*, *Streptococcus viridians*, *Enterococcus faecalis*, *Gardnerella vaginalis*, and *Prevotella bivia* [9,10,11]. Although in healthy women vaginal microbiota possesses a relatively low diversity, it should be considered that its patterns can undergo changes throughout the female lifecycle and the menstrual cycle [11] (see Table 1). 

### 2.2. Functions of Microbiota in the Vaginal Niche

In a similar manner to that of gut microbiota, vaginal microbiota could comprise an important modulator of inflammatory responses in the female genital tract [16]. In this regard, a study performed by Anahtar et al. [10] revealed high genital ecological diversity and low abundance of *Lactobacillus* in a group of asymptomatic young women from Africa [17]. Their findings also showed a correlation between high-diversity communities with genital pro-inflammatory cytokine concentrations. In addition, these authors suggested that genital antigen-presenting cells may sense Gram-negative bacterial products in situ via Toll-like receptor 4 (TLR-4), contributing to genital inflammation through activation of the NF-kB signaling pathway and recruitment of lymphocytes by chemokine production [17]. 

On the other hand, *Lactobacillus* species exert important health-promoting effects to maintain the reproductive fitness of the host and to maintain their dominance in the vaginal niche. This is accomplished by various direct and indirect antipathogenic mechanisms, such as the production of biochemically active compounds that directly kill or inhibit pathogens, and biophysical mechanisms, such as the formation of colonies that attach to the epithelial cells and form a physical barrier against pathogen adhesion, and the stimulation of host defense mechanisms against pathogens [16]. In this respect, *Lactobacillus* species produce lactic acid that acidifies the vaginal environment to a pH of 3.5–4.5, which has been shown to kill or inactivate a variety of vaginal pathogens, such as uropathogenic *Escherichia coli*, *Neisseria gonorrhoeae*, and *Chlamydia trachomatis* [16,18,19,20,21,22]. On the other hand, in vitro studies have revealed that several *Lactobacillus* species impede the attachment of pathogens to vaginal epithelial cells through their adhesion to host cells, preventing colonization by *E. coli*, *Gardnerella vaginalis*, *Klebsiella pneumonia*, *Pseudomonas aeruginosa*, *Staphylococcus aureus*, *group B streptococci*, and *Trichomonas vaginalis* [23,24,25,26,27]. 

### 2.3. Vaginal Dysbiosis and the Formation of Bacterial Biofilm

**Figure 1:**
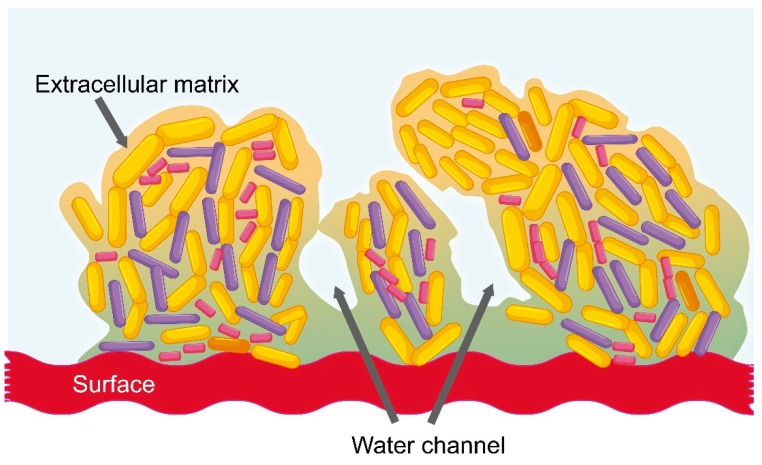
Proposed architecture of the biofilm as a mechanism of resistance of bacteria (see text for details).

In healthy women, vaginal microbiota is balanced; however, hormonal changes, douching, and sexual activity may imbalance it. This imbalance can promote vaginal colonization by potentially pathogenic microbes, typically causing bacterial vaginosis, aerobic vaginitis, and sexually transmitted infections, such as human immunodeficiency virus (HIV)-1, human papilloma virus (HPV) infection, and *Chlamydia trachomatis* infection [28,29,30,31]. Bacterial vaginosis is the most common form of vaginal infection in women of reproductive ages. It can be a chronic recurrent infection, which is typically characterized by the reduction of *Lactobacillus* and an overgrowth of *Gardnerella vaginalis* and other anaerobic bacteria, such as *Atopobium vaginae*, *Bacteroides* spp., *Mobiluncus* spp., and *Prevotella* spp. [28,32,33]. These anaerobic bacteria engage in synergistic interactions and may form a polymicrobial biofilm, which is considered one of the factors contributing to the chronicity and recurrence of the disease [28]. A bacterial biofilm is a structured group of bacteria adhered to an inert surface or a biological tissue (Figure 1) [34]. It possesses a sophisticated internal architecture and contains channels that permit the circulation of nutrients and divided areas that can contain genetically identical cells exhibiting different profiles of gene expression. This leads to an increased tolerance of unfavorable conditions, better persistence in adverse environments, and protection against antimicrobial molecules (e.g., antibiotics, antiseptics, etc.) and human immune responses [35,36].

The composition of the biofilm matrix is highly variable and it depends on surrounding environmental conditions [37]; however, one of the major virulence factors of bacterial biofilm is the production of extracellular polymeric substances (EPSs), because it decreases the penetration of antimicrobial molecules through its structure [36]. Likewise, EPSs have the ability to reduce the action of human immune response cells, such as neutrophils and macrophages [36]. The extracellular matrix (ECM) constitutes a physical barrier against all types of antibiotics, which may be constrained by the viscous matrix and be slowed down, causing decreased penetration into the biofilm [36]. Moreover, other biofilm environment-related factors, such as pH, pCO_2_, or pO_2_, may further affect the efficacy of the antimicrobials, e.g., aminoglycoside antibiotics lose antimicrobial activity under anaerobic conditions [38]. Likewise, bacterial cells reduce their metabolism inside biofilm, which inhibits the action of some antimicrobial molecules [38].

### 2.4. Current Treatment of the Most Common Vaginal Infections

According to the U.S. Centers for Disease Control and Prevention, bacterial vaginal infections must be treated with the following antimicrobial agents: metronidazole, tinidazole and clindamycin [39]. Metronidazole and tinidazole belong to the nitroimidazole class of drugs; these are antimicrobial agents used to treat trichomoniasis, amebiasis, and anaerobic bacterial infections. These drugs act by interfering with DNA synthesis [40]. Metronidazole is considered the drug-of-choice for treatment related to bacterial vaginosis; it is a first-generation nitroimidazole, which was initially indicated for the management of trichomoniasis, but was then shown to be effective against anaerobic microorganisms [41]. However, metronidazole therapy is associated with several adverse effects, such as nausea, vomiting, and gastrointestinal complaints [41]. Tinidazole is a second-generation nitroimidazole with a longer half-life than metronidazole (12–14 h vs. 6–7 h) and a better adverse effect profile [40]. The efficacy of tinidazole is similar to that of metronidazole, and a single-dose regimen is at least as effective as clindamycin vaginal cream [40]. On the other hand, secnidazole is a novel granular-formulation member of the family of 5-nitroimidazoles that has been approved for the treatment of bacterial vaginosis and trichomoniasis [40]. In vitro studies with secnidazole demonstrated clinical and microbiological evidence of activity against many anaerobic Gram-positive and Gram-negative bacteria implicated in bacterial vaginosis and limited activity against beneficial *Lactobacillus* species [40]. Finally, clindamycin is a lincosamide with similar efficacy to that of metronidazole; it prevents peptide bond formation, thereby inhibiting protein synthesis by reversible binding to 50S ribosomal subunits. Elsewhere, clindamycin is available in various pharmaceutical formulations including vaginal dosage forms and oral (systemic) pills [42]. However, when applied topically, clindamycin might weaken latex products such as condoms and may even cause pseudomembranous colitis [41].

## 3. Main Factors That Modify Vaginal Microbiota

As mentioned previously, vaginal microbiota plays a key role in preventing colonization by pathogenic organisms and maintaining the reproductive and gynecologic female health. However, there are various factors that could alter the vaginal microbiota, such as pathologies (e.g., aerobic or anaerobic vaginitis), pregnancy, age, diabetes, menopause, and even smoking. In this section, the main factors that modify vaginal microbiota will be described.

### 3.1. Aerobic Vaginitis

The vaginal disorder known as aerobic vaginitis was described in 2002 as a need to differentiate it from bacterial vaginosis [43,44]. In the first condition, the *Lactobacillus* microflora is disturbed, triggering an increase in the pH (between 6 and 8) and a yellowish or yellow-green homogeneous discharge is present. In addition, an increase in the numbers of intermediate and parabasal cells is observed, indicating increased turnover and desquamation of superficial epithelial-cell layers, which induce epithelial inflammation. As previously mentioned, *Lactobacillus* species perform an indispensable function in vaginal microbiota: inhibiting the growth of urogenital pathogens through the action of the surface proteins of *Lactobacillus crispatus* and *Lactobacillus jensenii*. Therefore, the diminution or absence of lactobacilli allows the proliferation of aerobic microorganisms, mainly group *B. streptococci*, *Staphylococcus aureus*, and *Escherichia coli* [45]. According to that reported by Donders et al. [43], in patients with aerobic vaginitis, *Staphylococcus aureus* was the most prevalent organism, followed by *Escherichia coli*. Interestingly, other authors [29] have reported that group *B. streptococci* and *Enterobacteriaceae* are the most frequent species in this pathological condition. These slight differences may depend on different factors, such as age, race, or sexual partners.

### 3.2. Bacterial Vaginosis

Bacterial vaginosis is a highly prevalent disease among women worldwide. It is characterized by a white-grey and homogeneous discharge (containing exfoliated epithelial cells and, attached to their surfaces, Gram-variable polymorphic bacteria), as well as a pH of ≥ 4.5 with a non-inflammatory process at the epithelium. This disorder is associated with severe changes in the composition of the vaginal microbiota, such as the diminution of *Lactobacillus* and colonization by anaerobic microorganisms, mainly *Gardnerella vaginalis*, *Prevotella* spp., *Atopobium vaginae*, *Bacteroides*, *Peptostreptococcus*, *Mycoplasma hominis*, *Sneathia*, *Leptotrichia*, *Mobiluncus* spp., and bacterial vaginosis-associated Bacterium 1 (BVAB1) to BVAB3 [46,47]. In this respect, some studies have demonstrated that species such as *Gardnerella vaginalis* were present in > 90% of symptomatic subjects, while these were present in < 45% of healthy subjects [48,49]. Likewise, the authors reported that *Lactobacillus* spp. was present in > 70% of normal subjects and in < 40% of symptomatic subjects. Moreover, it has been described that *Mobiluncus* was present in 40–60% of subjects with bacterial vaginosis [50]. As mentioned previously, in this disorder, a biofilm resistant to antibiotic therapies is usually formed on vaginal epithelial cells (denominated “clue cells”), which increases the persistence and recurrence of infection [29]. Interestingly, this disease has been associated with sexually transmitted infections such as HIV and HPV; for this reason, bacterial vaginosis has emerged as a public health priority.

### 3.3. Pregnancy

In addition to endocrine, metabolic, and immune alterations, there are important changes in the microbiota during pregnancy, such as a decrease in overall diversity and enrichment with *Lactobacillus* species. Freitas et al. [51] carried out a comparative study between pregnant and non-pregnant women. These authors reported that the vaginal microbiota of healthy pregnant women presented a lower concentration of *Mycoplasma* and *Ureaplasma* and a higher bacterial load compared with those of non-pregnant women. Furthermore, the authors found a higher prevalence of *Lactobacillus* species in pregnant women than in those found in non-pregnant women. Similarly, Romero et al. [52] used sequence-based methods and found that *Lactobacillus* spp. comprised the predominant members of vaginal microbiota in pregnant women. Remarkably, the changes in vaginal microbiota during pregnancy were associated with a decrease in the vaginal pH and an increase in vaginal secretions [53,54]. As previously mentioned, *Lactobacillus* species present bactericidal activity; thus, increases in their presence could represent a protection mechanism against infection during pregnancy [54]. 

### 3.4. Age 

**Figure 2:**
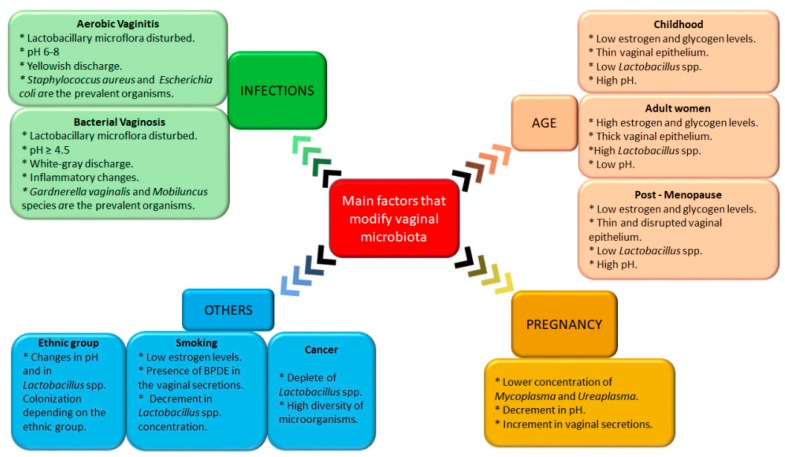
Main factors that modify vaginal microbiota. Different factors could change the composition of vaginal microbiota, triggering modifications in this area. Some of these factors include the woman’s age, pregnancy, ethnic race, presence of cancer, and smoking habit, among others.

During the maturation of women, changes in the composition of vaginal microbiota are produced; these changes are strongly related to estrogen levels. In childhood, the vaginal environment presents a slightly alkaline (or neutral) pH and microbiota is mostly composed by Gram-negative anaerobic bacteria such as *Veillonella*, *Bacteroides*, and *Fusobacteria*, among others, and Gram-positive anaerobic bacteria including *Actinomyces*, *Peptococcus*, and *Peptostreptococcus* [12,13,55]. Likewise, in prepuberal girls, a low frequency of *Lactobacillus* spp., *Gardnerella vaginalis*, *Prevotella bivia*, and *Mycoplasma hominis* is observed, as well as low levels of estrogen and glycogen, which correlate with a thin mucosa. Maturation of the vaginal epithelium during puberty is achieved by the deposition of glycogen in this epithelium, a process mediated by estrogen levels (Figure 2). As estrogen levels increase during puberty, glycogen deposition in the vaginal epithelium increases to the same proportion, permitting the ascendance of lactic acid-producing bacteria. These bacteria ferment glycogen into glucose and eventually into lactic acid, which results in a decrease in pH that establishes an inhospitable environment for pathogens, preventing infections and protecting the genital tract [56,57]. Adolescent girls present a vaginal microbiota similar to that of adult women, in which *Lactobacillus* spp. predominate [58]. In fact, more than 20 *Lactobacillus* species have been detected in the vagina; however, previous reports revealed that there are not high numbers of many different species, but that one or two lactobacilli from a range of three or four species are dominant, primarily *Lactobacillus crispatus*, *Lactobacillus iners*, *Lactobacillus jensenii*, and *Lactobacillus gasseri*. An environment rich in *Lactobacillus* species is associated with vaginal health, because these, in addition to maintaining an adverse environment to many harmful bacteria, produce H_2_O_2_, toxic hydroxyl radicals, bacteriocins, and probiotics. 

On the other hand, estrogen levels in women decrease as the latter reach menopause; during the reproductive years, the concentration of estrogen in plasma is approximately 129 ng/L, while after menopause, the plasma content is about 18 ng/L [59]. Thus, the amount of glycogen in the vaginal epithelium is reduced and consequently, lactobacilli dominance is decreased. As in prepuberal girls, less lactic acid is produced due to the low lactobacilli concentration, which results in an increase in vaginal pH [56,60]. The alkalization of pH in vaginal microbiota permits potential pathogens to invade or expand their colonization in the vagina. In agreement with this, some reports showed that, in an alkaline environment, the vagina is colonized mainly with fecal flora such as *Enterobacteriaceae*. Moreover, low estrogen levels in postmenopausal women produce structural changes such as dryness, loss of elasticity, and a decrease in blood flow [61,62].

### 3.5. Others

There are other factors that could modify vaginal microbiota, such as smoking, ethnic group, and the presence of cervical cancer. It has been reported that smoking is highly related to vaginal microbiota lacking in the protective action of *Lactobacillus* spp. In 2014, Brotman et al. [63] performed an evaluation of the vaginal microbiota of 20 smoking and 20 non-smoking women. These authors found that the concentration of *Lactobacillus* spp. exhibited a decrease in smokers’ vaginal microbiota compared with that of non-smokers. In addition, other studies have suggested that smoking may predispose women to bacterial vaginosis [64,65]. This increased susceptibility is probably due to the anti-estrogenic effect of smoking, and to the presence of benzo[a]pyrene diol epoxide (BPDE) in the vaginal secretions of smokers, which has been reported to increases bacteriophage induction in *Lactobacillus* spp. [63].

In recent years, diverse authors have reported that the composition of vaginal microbiota is highly related to the woman’s ethnic group. For example, Ravel et al. [9] evaluated the differences in the vaginal microbiota of 396 apparently healthy women of North America, representing four ethnic groups (White, Black, Hispanic, and Asian). These authors found that the dominant vaginal bacterial communities in Asian and White women (80.2% and 89.7%, respectively) included *Lactobacillus crispatus*, *Lactobacillus gasseri*, *Lactobacillus iners*, and *Lactobacillus jensenii*, while for Hispanic and Black women, the dominant communities were not *Lactobacillus* spp. This behavior could be related to differences in pH, in that Hispanic and Black women presented higher pH (5.0 ± 0.59 and 4.7 ± 1.04, respectively) compared with Asian and White women (4.4 ± 0.59 and 4.2 ± 0.3, respectively). Similarly, Zhou et al. [66] found significant differences in the frequencies of bacterial types between asymptomatic Japanese women and White and Black women from North America. These authors reported that lactobacilli communities present an incidence of 24.7% in Japanese women, while, in Black women, this incidence was 40.5%, and in White women 13.3%. Additionally, the dominant lactobacilli communities were common in both Japanese and White women but were not in Black women.

**Figure 3:**
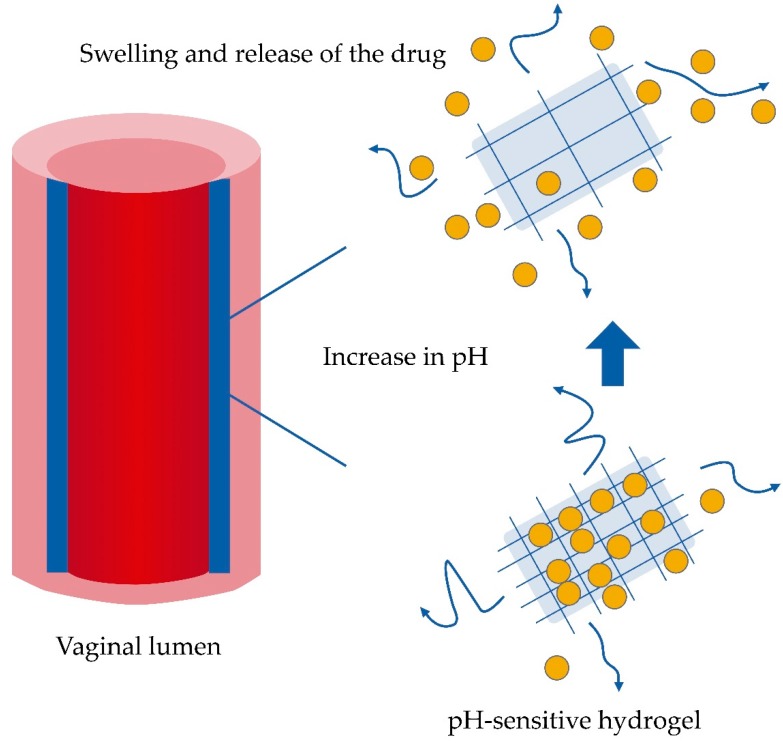
Deposition of a hydrogel on the vaginal wall for the release of drugs. The swelling ability and dissolution time of polymers are the basic mechanisms that control the release of a drug toward the vaginal wall or lumen. Smart polymers such as pH-sensitive hydrogels are able to modulate drug release by swelling under pH modifications.

On the other hand, to date, the relationship between vaginal microbiota and the presence of cervical cancer has not been clarified. Some authors found that suppression in the quantity and activity of *Lactobacillus* was related to the development of cervical cancer [67]. Similarly, the deficiency of an antioxidant mechanism (that compromises the vaginal microbiota) and an inefficient genetic profile could contribute to the initiation and progression of cervical cancer [68]. In 2015, Mitra et al. [69] compared the vaginal microbiota of women with pre-invasive cervical intraepithelial neoplasia (low and high), invasive cervical cancer, and healthy women. They found an increase in the diversity of vaginal microbiota (and depleted *Lactobacillus* spp.) depending on the disease grade. Vaginal microbiota impacts the physiological functions of vaginal epithelial cells in order to maintain the integrity of the barrier that they form; thus, when the homeostasis barrier fails, a favorable environment for carcinogenesis is created [68]. As can be observed, there are different factors that can change the composition of vaginal microbiota. Figure 3 depicts these factors and their main characteristics.

## 4. Physicochemical Changes Produced by Modification in Vaginal Microbiota

Variations in the vaginal environment caused by alterations in the local microbiota consist predominantly of changes in pH, viscosity, and fluid composition or discharge, among others. As previously noted, the vaginal environment maintains its stability by means of the actions of commensal organisms such as *Lactobacillus*; the normal vaginal pH falls within the range of 3.8–4.5 in menstruating females, due principally to that *Lactobacillus* species produce substances that lower the pH of the vaginal environment. However, pathological conditions such as bacterial vaginosis or vulvovaginitis produce increases in pH [70,71]. It is noteworthy that, in addition to changes in *Lactobacillus* colonization, there are certain other conditions, such as oxygen restriction and high carbon dioxide, which contribute to the acidity of the cervicovaginal fluid; these conditions may also be influenced by changes in vaginal microbiota [22]. With respect to viscosity and the composition of the vaginal fluid, the physiological discharge in the reproductive-age woman must be a thin-to-pasty liquid and, depending on the phase of the menstrual cycle, clear, white, or light gray [72]. Fluctuating levels of estrogen and progesterone during the menstrual cycle greatly affect the consistency and composition of the physiological discharge. In addition to lactobacilli, other commensal organisms found in the vagina include streptococci, enterococci, and a few other organisms that form part of the normal flora, but that are associated with vaginal infections, including anaerobic *Bacteroides*, anaerobic cocci, *Gardnerella vaginalis*, and *Candida* [73]. In this respect, during an infectious period and depending on the severity of the changes in the microbiota, the clinical description of vaginal discharge changes and the discharge tends to be homogeneous, purulent, yellowish or yellow-green in color, and with a fishy or rotten odor [47].

## 5. Effects of Vaginal Microbiota on the Metabolism of Drugs

It is traditionally thought that individual patient responses to therapeutic agents are only modulated by genetic polymorphisms and drug–drug interactions; however, evidence has shown that a third factor that may modulate the effects of exposure to drugs is the human microbiota [74]. Concerning this, topical drugs such as tenofovir, a vaginal antiretroviral gel, can be metabolized by certain vaginal species [75]. A recent study in African women described the role of *Gardnerella vaginalis* in the metabolism of tenofovir [76]. In that study, a difference in the therapeutic efficacy of the antiviral was found. Tenofovir exhibited higher effectiveness in women with *Lactobacillus*-dominant microbiota and HIV incidence was reduced by 61%, whereas, in women with predominant *Gardnerella vaginalis*, the incidence of reduction reached only 18%, a 3-fold difference in efficacy [76]. The authors concluded that *Gardnerella vaginalis* decreased the therapeutic action of tenofovir through increasing its metabolism. On the other hand, it is feasible to speculate that, due to that, vaginal microbiota undergoes modifications during the lifecycle of women. The efficacy of tenofovir could change depending on the age of the patients.

On the other hand, statins have shown less effectiveness in reducing low density lipoproteins in African women than in European women [77]. It has been demonstrated that African women and European women exhibit differences in vaginal microbiota; thus, the participation of vaginal microbiota in these effects cannot be ruled out.

Finally, vaginal microbiota could control the efficacy of drugs indirectly by altering the host metabolism and producing metabolites that compete with the drug receptors [78]. In support of this hypothesis, Nelson et al. [65] used diverse approaches in order to demonstrate that bacterial composition was the major factor determining the vaginal metabolome. 

## 6. Possible Effects of Changes in Vaginal Microbiota on Drug Release—Challenges and Opportunities

### 6.1. Influence of pH, Viscosity, Fluid Composition, Drug Metabolism, and other Factors on Drug Delivery

Among different mucosal routes for delivering drugs, the vagina represents an excellent anatomical organ for drug administration under gender-specific conditions for both local and systemic effects. Above conventional drug-delivery systems, vaginal drug delivery possesses the following main advantages [3,79]:

- Ability to bypass first-pass metabolism
- Easy handling, in that self-insertion and/or removal can be performed by patients
- High permeability for low molecular weight drugs
- Relatively large surface area for absorption
- Rich blood supply
- Less potential for pain, tissue damage, or infection compared to parenteral routes.

However, as with other routes, the effectiveness of the vaginal route depends on intrinsic factors of the application site, such as pH, fluid composition, viscosity, enzymatic metabolism, clearance, etc. In this regard, the pharmacokinetics of many drugs is modulated by their ionization state, which is defined by the pH at the administration site. In the particular case of the vagina, the pH ranges from 3.8–4.5 in healthy women, and it increases in infections. In the universe of drugs, about 95% of drugs possess an ionizable group, and of these, 75% are weak bases [80]. In the case of drugs intended for vaginal administration, at the vaginal pH, the majority of the weak bases are found in the ionized form (more than 50% of the weak bases have a pKa = 8.5–10.5) [80]. In the case of weak acids, about 40% have a pKa < 5.5 [80]; this means that they will be found un-ionized at the vaginal pH. From these data, we can infer that weak acid drugs possibly would have better absorption than weak bases at a normal pH; however, presumably the absorption profile may change if the pH is modified in certain illnesses. Concerning this, a few researches have explored the effect of pH on drug performance. For instance, the effect of the vaginal pH over the induction of labor with misoprostol and dinoprostone was studied by Chandra et al. [81] and by Kurian et al. [82], respectively. No effect was observed when misoprostol was employed (pKa = 14.68), while the pH was a positive factor for the effectiveness of dinoprostone (pKa = 4.9). 

In healthy women, vaginal fluid is constituted of cervical secretion and transudation from blood vessels, desquamated vaginal cells and leukocytes; it also contains enzymes, proteins, carbohydrates, amino acids, and other molecules [79,83]. The volume and viscosity of the vaginal fluids may also affect the drug release and absorption of the drugs; for example, drugs must be soluble to be absorbed, and the solubility of a drug is intrinsically related to the quantity of solvent available for solubilization. In vaginal fluids, water available for solubilization is affected by the menstrual cycle and changes in the microbiota, which may cause variability in the response of the drugs. Although any drug intended for vaginal delivery requires a certain degree of solubility in water, the absorption of a drug that is poorly water-soluble may be increased when the fluid volume is higher [79]. Thus, if there is a large volume of vaginal fluid, then low hydro-soluble drugs will dissolve effectively, but this increases the feasibility of the drug being expelled due to gravity [84]. 

On the other hand, vaginal mucus acts as a lubricant and protection barrier against pathogens and other harmful substances; it functions as a permeable gel layer where the exchange of nutrients and gases to and from the basal epithelium takes place [83]. Despite its pivotal role, the viscosity of the mucus can be an obstacle to the diffusion of the drug from the formulation or drug-delivery system: the higher the viscosity, the lower the rate of release. However, too low a viscosity (thick mucus) may increase the removal of the formulation and decrease the bioavailability of drugs administered via the vaginal route. Under some health conditions and disorders, the quantity and viscosity of the mucus is altered; thus, it is imperative for the dosage form to adhere to the vagina during a sufficient time of residence in order for it to exert its therapeutic effects. This can be achieved by utilizing mucoadhesive polymers or materials with gelling ability (see Section 6.2 for more information). 

Recently, novel nanostructured drug-delivery systems have been developed for vaginal administration to achieve systemic action. However, it is important to focus on vaginal mucus as a barrier and a clearance mechanism that can limit the vaginal retention of nanoparticles [1]. Vaginal mucus is a complex biphasic fluid composed of a low molecular weight component (cervical plasma) and a high-molecular-weight component (gel phase) [85]. Thus, the composition of the mucus and its variations due to hormonal cycles, age, or illness can limit nanoparticle distribution, diffusion, and their ability to effectively cross deepest into the epithelium layers. Disruption of the mucus can allow nanoparticles to penetrate rapidly, improving vaginal drug distribution and retention; the surface coating of nanoparticles with densely packed polyethylene glycol has permitted the development of mucus- penetrating particles with improved delivery characteristics [86]. The key to achieving mucus-penetrating systems is to avoid interaction among nanoparticles with proteins and protein-rich surfaces. To our knowledge, there are no studies on how the microbiome modulates the adhesion or penetration into the cervical mucus of nanoparticles. Nonetheless, but at first instance, the excessive production of glycoproteins by vaginal microbiota to form a protective biofilm should increase the bioadhesion of mucoadhesive conventional nanoparticles, limiting their passage through the epithelium.

Despite the vaginal route being considered a manner of administration involving relatively low enzymatic activity, this aspect should be seriously examined, mainly when peptides are employed as drugs. The vagina’s external cell layers and basal cell layers retain the majority of the enzymatic activity because, at this site, the following can be found: aminopeptidases; dipeptidyl peptidases, and dipeptidyl carboxypeptidases. From a pharmacokinetic point of view, aminopeptidases play an important role in the degradation of peptide and protein drugs in the vagina; thus, increases in enzymatic activity may considerably affect drug absorption [87]. In this regard, during infections such as bacterial vaginosis, the associated bacteria also produce enzymes capable of degrading peptide drugs or of inducing changes in the consistency of the mucus. Olmsted et al. [88] found an increase in the glycosidase and proteinase activity of anaerobic Gram-negative bacteria. In addition, clinical observations demonstrated that vaginal fluid samples from women with bacterial vaginosis were less viscous than samples from women with normal microflora, the latter due to the increase in enzymatic activity. 

All of the changes mentioned herein could modify drug-release profiles during treatment; therefore, formulators should take into account the particularity of the vaginal route in order to develop drug-delivery systems that adapt to the changing conditions of the vaginal fluids due to the hormonal cycle, age, and pathologies. For example, compared with the intestinal epithelium, the metabolic activity of the vaginal epithelium is lower; thus, different materials should be employed to constitute an appropriate pharmaceutical dosage form to avoid degradation of the drug and a reduction in its therapeutic action, especially in disorders where the vaginal epithelium’s normal function is compromised. 

### 6.2. Use of Smart Excipients to Take Advantage of Microbiota Modifications and Improve Drug Efficacy

Vaginal administration of drugs has been directed mainly for the treatment of local effects related to infections and for contraceptive purpose [89]. However, the vaginal route for drug administration allows the use of prolonged-release systems, avoiding the need to administer daily doses. In addition, systemic effects can also be achieved through this route of administration, with a relevant characteristic: it is a reversible administration route [90]. For vaginal purposes, the application of hydrogels has been attractive due to the ease of manufacturing, cost-effectiveness, and simplicity of application. Hydrogels are three-dimensional cross-linked networks of polymer chains that absorb and conserve water in the interstitial spaces between the chains [91]. New developments have been relevant for the incorporation of different matrix systems into the nanometric range with hydrogels to improve biopharmaceutical results. In addition, obtaining new polymers has also allowed improving the performance of drugs. The application of stimulus-sensitive formulations to pH, as a complementary antibiotic treatment, for example, for a condition of bacterial vaginosis, is attractive because it would control any change deriving from a relapse in the infection. pH-sensitive hydrogels are designed to undergo a volume phase transition, such as collapse and swelling. This transition modulates the dissolution of the drug [91]. pH-sensitive hydrogels are determined by the pH and the nature of the exposed functional groups, the ionic charge, the pKa of ionizable groups, and the degree of ionization, hydrophilicity, and polymer concentration. The formulation of pH-sensitive hydrogels can be achieved with polymers such as chitosan, guar gum, carrageenan, dextran, xanthan gum, cellulose, alginate, poly(acrylic acid), poly(acrylamide), poly(vinyl alcohol), poly(vinyl pyrrolidone), and poly(lactic acid), or their combinations. It is noteworthy that anionic hydrogels could be more useful for the release of antibiotics into acidic media for the vaginal area. With basification of the vaginal environment during a change in vaginal microbiota, the protonated negatively charged moieties on the polymer chains give rise to repulsion and then swelling. In the case of anionic polymers, a pH above the pKb produce ionization of the functional groups and an increase in the hydrophilicity of the polymer, which allow a greater uptake of water, relaxation of the polymer chains, and greater diffusion of the drug toward the external environment (Figure 3) [91]. Anionic polymers are generally controlled by ‒COOH groups. Therefore, in bacterial vaginosis, an increase in pH produced by decreases in the concentration of lactic acid and hydrogen peroxide may be the factor that stimulates the release of the drug. Inversely, once the infection ceases and the microbiota returns to its normal level of acidosis, release of the active molecule could decrease. More complex and possibly also more effective formulations may include the composition of pH-sensitive hydrogels and matrix systems in nanometric sizes. For example, an anionic polymer that responds to a change in pH toward the basic level by exceeding its pKb will begin to swell by means of the ionization of its functional groups, and to release a nanoparticle system that incorporates a drug inside, for example, an antibiotic. This alternative is especially attractive when the bacterial biofilm has been formed and different internalization systems are required. The controlled deposition of nanoparticles by a pH change would create a concentration gradient toward the interior of the biofilm, and diffusion would be favored by small particle sizes and the mimicry of polysaccharides [35]. Inevitably, the degree of swelling would also be influenced by the ionic strength of the vaginal fluid.

Regardless of the hydrogel drug carrier assembly, the same nanocarrier has been used under different conditions such as pH-sensitive and muco-adherent systems. However, equivalence in the administration of the dose can be a limitation for low-potency drugs when they are systems with low encapsulation efficiency and even a moderate percentage of drug loading. An alternative in the search for local treatments is the use of microcarrier systems, which allows greater incorporation of the drug. Furthermore, systems can become more sophisticated, such as the assembly of nanoparticles into microparticles embedded in a muco-adherent hydrogel. In this proposal, the hydrogel is pH-sensitive, the pH-sensitive microparticle has a different degree of response, and finally, the drug is inside the bio-adherent nanoparticle, or a first drug is released from the microparticles, and a second from the nanoparticles. In the case of vaginosis and biofilm formation, the ternary systems would allow the deposit of the hydrogel and the microparticles on the biofilm surface, and the nanoparticles would internalize until they achieve close contact with bacterial microcolonies. The main challenges include having the therapeutic dose required, in the necessary time, in the right place, without these being modified by the different factors of the vaginal microenvironment.

### 6.3. Feasibility of the Use of Hydrogels to Control the Release of Drugs in Vaginal Microbiota Changes

Given the various modifications in the vaginal environment, maintaining a constant drug effect becomes a challenge [92]. Under specific and well-known conditions of alterations in the vaginal microbiota, even the design of personalized medications becomes necessary [3]. In this respect, the pharmaceutical forms that permit greater manipulation for personalized medications are hydrogels, because a hydrogel can absorb the majority of the variations of the vaginal environment to give way to releasing systems such as nanoparticles [93]. Even a hydrogel that is not formulated to be sensitive to a stimulus can be an attractive alternative in the assembly of other controlled-release systems. Therefore, an increasing number of commercial drugs for the treatment of vaginal infections are being formulated as hydrogels [94]. On the other hand, some aspects traditionally sought in a hydrogel for vaginal administration include mucoadhesion and a high retention time, as well as biodegradability and biocompatibility. Thus, to solve the changes in vaginal microbiota and to achieve a desired therapeutic effect, traditional hydrogels may be physically combined with other polymers to improve their rheological and mucoadhesion properties. In this regard, hydroxypropil methylcellulose and chitosan are two highly mucoadhesive polymers with strong potential usefulness. In addition, there is a wide range of new opportunities for obtaining new excipients from techniques such as freeze-thawing and gamma radiation. It should be noted that, although these methodologies involve extensive biosecurity studies, the results are usually promising, especially when the novel polymer is obtained by mixing natural and synthetic polymers. In any of these cases, for systems in hydrogels, rheology studies are also essential for deposition into the vagina from the applicator and for understanding the variations of the hydrogel according to the anatomical movement, for example, during coitus. The combination of different monomers sensitive to different factors also permits the obtaining of multi-responsive polymers, for example, pH and temperature [95]. In addition, a relative control in the assembly of the new polymer can be provided in order to confer self-assembly and form various nanosized structures. Self-assembly behaviors not only control the release but also the interaction with the biological environment, and can promote greater or lesser internalization in tissues according to the desired objective.

In the context of the information described in this document, more research in the area is necessary to have defined objectives in the development of new medicines. The impact of the human microbiome on our metabolism and its relationship with different diseases has changed the description of some pathogeneses, therefore the conception of novel drugs. This is a huge area of research that can continue its investigation with the interaction of normal vaginal microbiota and the drugs destined for this route of administration in terms of solubility and biotransformation, and the influence of vaginal microbiota on the excipients and on the excipient-drug relationship. In addition to the variations in pH and viscosity deriving from alterations in the microbiota on the drug, excipient, and excipient-drug. Better knowledge of this research route will lead to the provision of drugs with better efficacy or to the formulation of personalized medication.

## 7. Conclusions

The vaginal cavity represents an attractive alternative for delivering local and systemic treatments due to its high vascularity and the avoidance of first-pass effects; however, diverse factors may produce alterations in vaginal microbiota. Although this microbiota plays a major role in maintaining the health state of the female reproductive tract, a microbiota imbalance may produce a series of changes in the vaginal environment, altering the composition of fluids, the pH, enzymatic activity, and viscosity, which would lead to altered drug absorption. Therefore, maintaining steady drug delivery, thus a steady therapeutic effect, is a strong challenge. Concerning this, formulations such as hydrogels could be the ideal pharmaceutic form for this purpose. These can be designed with unique properties, conferring on it the ability to resist severe variations in the vaginal environment. However, further investigation is needed to support this hypothesis.

## Figures and Tables

**Table 1:** Predominant microorganisms throughout the female lifecycle.

Woman’s lifecycle	Predominant Microorganisms	References
Childhood	**Gram-negative anaerobic bacteria**, such as *Bacteroides, Fusobacterium, Veillonella* **Gram-positive anaerobic bacteria**, such as *Actinomyces, Bifidobacterium, Peptococcus, Peptostreptococcus, and Propionibacterium* **Aerobic bacteria** such as *Staphylococcus aureus, Staphylococccus epidermidis, Streptococcus viridans,* and *Enterococcus faecalis*	[12,13]
Prepuberal	Low abundance of *lactobacilli, Gardnerella vaginalis*, and *Prevotella bivia*	[13]
Puberty	Predominant species are *Lactobacillus crispatus, Lactobacillus gasseri, Lactobacillus iners*, and *Lactobacillus jensenii*	[14]
Adult	Similar to puberty, *Lactobacillus crispatus, Lactobacillus gasseri, Lactobacillus iners*, and *Lactobacillus jensenii*	[14]
Menopause	Predominant species are *Lactobacillus crispatus, Lactobacillus iners, Gardnerella vaginalis*, and *Prevotella* and a lower abundance of *Candida, Mobiluncus, Staphylococcus, Bifidobacterium*, and *Gemella*	[15]
